# Desert Plant Trait Networks Differ Among Plant Life Forms and Soil Moisture–Salinity Environments

**DOI:** 10.3390/plants15172622

**Published:** 2026-08-27

**Authors:** Jifen Yang, Xueni Zhang, Yufei Chen, Huixia Li, Mengna Xu, Judu Hu

**Affiliations:** 1 College of Ecology and Environment, Xinjiang University, Urumqi 830017, China; 2 Xinjiang Biomass Solid Waste Resources Technology and Engineering Center, College of Chemistry and Environmental Science, Kashi University, Kashi 844000, China; 3 Key Laboratory of Oasis Ecology of Ministry of Education, Xinjiang University, Urumqi 830017, China; 4 Xinjiang Jinghe Observation and Research Station of Temperate Desert Ecosystem, Ministry of Education, Urumqi 830017, China

**Keywords:** plant trait network, central traits, soil water–salt environment, adaptation strategy, functional traits

## Abstract

Understanding the relationships among plant functional traits and how these are shaped by the soil environment is essential for the study of plant adaptation. Conducted in the desert ecosystem surrounding Ebinur Lake, China, this study assessed key functional traits for eight common desert plant species, including the foliar carbon (C), nitrogen (N), and phosphorus (P) content, and their stoichiometric ratios (C:N, C:P, N:P), as well as the chlorophyll content (Chl), leaf area (LA), and leaf thickness (LT). Separate plant trait networks (PTNs) were constructed for herbs, shrubs, and trees growing in two distinct soil environments: those with high (HS) versus low (LS) soil moisture and salinity. The study had three key findings. (1) Both soil properties and plant functional traits differed between HS and LS environments (*p* < 0.05). (2) Distinct PTNs were observed among plant life forms, with different central traits. Herbs (central traits: leaf thickness and the N:P ratio) had high-density PTNs demonstrating strong adaptability, while shrubs (central traits: C and P) and PTNs had high connectivity that was stable across soil environments; tree PTNs (central traits: N and the C:P ratio) were highly modular and showed strong resistance to disturbance. (3) Soil factors also regulated PTN topology, with the total nitrogen (TN) and total phosphorus (TP) being key regulators in HS, while the soil salt content and water content were the most important factors in LS. In conclusion, the PTNs of desert plants reflected their unique adaptations to arid environments, and the application of these findings should support efforts to conserve and manage desert vegetation.

## 1. Introduction

Plant functional traits represent a comprehensive manifestation of an individual’s ability to acquire resources and adapt to specific environments [1,2], and are therefore widely used to evaluate plant adaptation. The adaptive mechanisms and growth strategies utilized by plants rely on the interactions among many functional traits [3]. As such, trait interactions have become a major focus in studies of plant adaptation and how plants respond to disturbance [4,5]. Long-term environmental adaptation has enabled desert plant species to develop robust tolerance to multiple stresses, such as drought and soil salinity, with this tolerance arising from synergistic interactions between morphological and chemical traits [6]. Exploring the relationships among functional traits to elucidate plant drought adaptations has become a key research focus, given the need to understand the impacts of global change on arid desert ecosystems [7].

Desert plants typically grow in environments exposed to drought, soil salinization, and nutrient scarcity. The vegetation in desert ecosystems is dominated by sparse xerophytic shrubs, with communities primarily composed of small trees, shrubs, and semi-shrubs [8]. Plants of all life forms use distinctive trait combinations and other drought adaptations to cope with environmental stressors in arid environments [9]. In a recent study of the most common small trees and shrubs in the dry desert regions of northwest China [10], key functional traits were more strongly correlated in small trees than shrubs, likely due to the more severe drought stress experienced by small trees. This suggests that plants adapt to changing environments not only by modifying a variety of traits but also by modifying the interrelationships among these traits [11].

Functional trait associations are critical for deciphering how plants respond to environmental change, as they reflect plant resource allocation strategies and stress-tolerance mechanisms [9,12]. Trait correlations provide information about trait co-optimization, trade-offs, and/or allometric relationships based on biomechanical and/or physiological requirements [13]. Indeed, many such trait relationships have proven common, or even ubiquitous, across diverse sets of species within and among communities and biomes, and even across global databases [14]. To study such trait associations, trait networks represent a promising approach that can accommodate complex multivariate relationships (such as those underlying adaptive plant strategies), making such networks better able to dissect the intricate interactions among functional traits than bivariate relationship-based methods [15,16]. Plant trait networks (PTNs) are mathematical models that represent plant traits as nodes connected by edges (statistical correlations between traits); network topology may be characterized by parameters such as complexity (modularity), connectivity (edge density), and node centrality (degree). These networks provide comprehensive and intuitive representations of trait correlations and trade-offs, and have been successfully applied to understanding environmental adaptations in plants [11]. For example, a recent study of fine root traits in arid ecosystems showed that PTN topological structure (i.e., edge density and modularity) reflected plant drought adaptation strategies [7]. Similarly, leaf trait networks for nine forest regions in China, spanning from tropical to cold temperate zones, differed among plant growth forms, suggesting that different growth forms employ distinct strategies to cope with local environmental conditions [17].

Plant trait network analysis has been extensively applied to explore forest dynamics and how large aquatic plants respond to environmental change [18,19,20], particularly to resource pulses. Biogeochemical cycles in desert ecosystems are generally slower than those in forest and aquatic ecosystems, leading to soil aridity and low net primary productivity [21]. Arid ecosystems are therefore highly sensitive to environmental change, as illustrated by functional trait networks constructed for plants in China’s desert ecosystems [22]. Although many studies have documented drought-tolerance strategies in desert plants [21,23,24], how PTN architecture diverges along soil moisture–salinity gradients remains poorly understood. Soil moisture and salinity constitute dominant abiotic filters shaping covariation among desert plant traits [25], and spatiotemporal variation in both soil factors strongly moderates PTN complexity and connectivity. Furthermore, distinct morphological and physiological attributes across plant life forms drive pronounced discrepancies in PTN responsiveness to combined drought–salinity stress [26]. As such, comparing PTNs along soil moisture–salinity gradients and among plant life forms is critical for understanding species-specific adaptive strategies in desert plants experiencing combined drought and salinity stress.

The Ebinur Lake Wetland National Nature Reserve is located in a typical desert region of the Junggar Basin in northern Xinjiang, China. Desert vegetation, such as that within the Ebinur Lake Reserve, is particularly sensitive to environmental change, with soil moisture and salinity being key abiotic factors affecting plant distributions and survival [8]. Therefore, in this study, soil moisture and salinity data were obtained for the Ebinur Lake Reserve, as well as measurements of core leaf functional traits widely used in desert plant ecology, including leaf morphology, chlorophyll content, and C:N:P stoichiometry. These traits are sensitive to drought and salt stress and are closely related to plant resource acquisition and stress tolerance [14]. Trait networks were constructed for herbaceous plants, shrubs, and trees to address the following questions: (1) What are the central traits and key topological structures of PTNs for different life forms under contrasting soil moisture–salt conditions?; (2) How does trait network structure, stability, and plasticity differ among herbs, shrubs, and trees?; and (3) How do soil moisture, nutrient availability, and salinity jointly affect PTN parameters and further regulate adaptive trade-offs between network plasticity and stability for plants of different life forms?

## 2. Results

### 2.1. Soil Factors and Plant Functional Traits Differed Between High and Low Salt-Stress Environments

Two contrasting soil environments were compared across the study area by dividing sampling sites into those with relatively high soil moisture levels and salinity (representing “high soil water–salt environment” where plants experience significant salt stress) and those with lower soil moisture levels and salinity (“low soil water–salt environment”) as assessed via a k-means clustering analysis. The soil water content (SWC), soil salt content (SSC), soil organic carbon (SOC), and total nitrogen (TN) were significantly higher in the high versus low soil water–salt environment (*p* < 0.05), whereas the soil total phosphorus (TP) did not differ between the two environments. The SWC and SSC were more than doubled in the high versus low soil water–salt environment (Table 1).

Eight common desert plant species, including herbs, shrubs, and trees, were surveyed within the study area (Table 2); these species displayed different habitat preferences and varied in abundance between the two soil water–salt environments. Among the herbaceous species, *Phragmites australis* occurred at high abundance in both environments, while *Glycyrrhiza uralensis* preferred high water–salt environments. Shrubs similarly showed different preferences, with *Apocynum venetum* common in both environments and *Halimodendron halodendron* restricted to high water–salt environments. The only tree sampled, *Populus euphratica,* had low overall abundance. These differences among species reflect clear differentiation in water–salt adaptation strategies.

A number of functional traits were measured for all study plant species (Table 3). Except for the chlorophyll content (Chl), leaf area (LA), and leaf thickness (LT), the measured plant functional traits differed significantly between high and low soil water–salt environments. The foliar carbon content, nitrogen content, C:P ratio, and N:P ratio were significantly higher in high versus low soil water–salt environments (*p* < 0.05); in contrast, the leaf phosphorus content and C:N ratio showed the opposite pattern (*p* < 0.05).

### 2.2. Plant Trait Networks Differed Among Life Forms and Soil Environments

**Figure 1:**
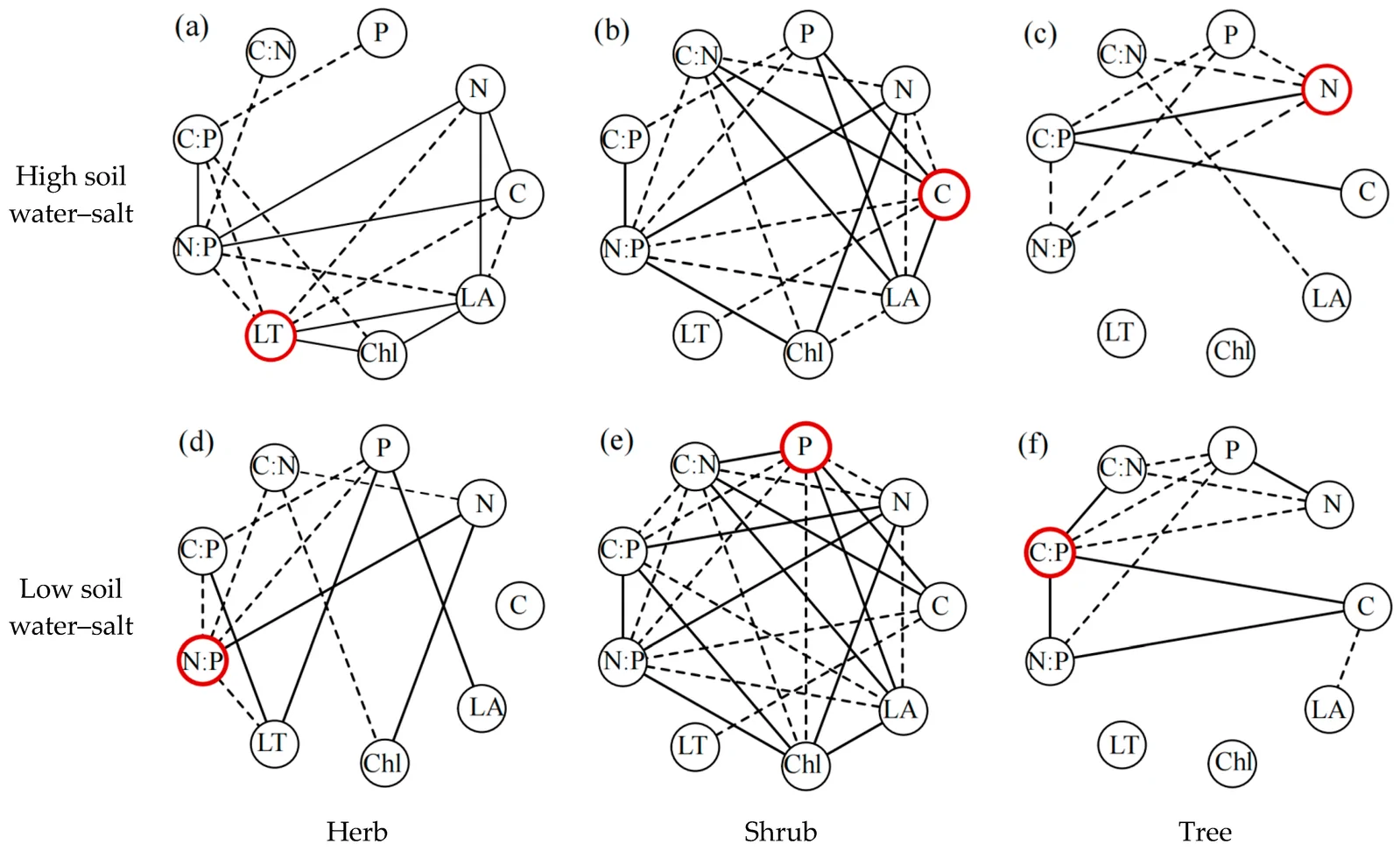
Plant trait networks for herbs (**a**,**d**), shrubs (**b**,**e**), and trees (**c**,**f**) for the high (**a**–**c**) and low (**d**–**f**) soil water and salt environments. Nodes represent functional traits. Solid lines indicate positive correlations between traits, while dashed lines indicate negative correlations. Nodes highlighted in red are the central traits of each trait network.

For both the high and low soil water–salt environments, plant trait network (PTN) complexity increased sequentially from trees to herbs and then shrubs (Figure 1). The central traits identified for each PTN also differed among plant life forms, although these were generally focused on foliar carbon (C), nitrogen (N), and phosphorus (P), as well as their stoichiometric ratios. The only exception was LT, the central trait selected for the herb PTN in a high soil water–salt environment.

Regarding connections among functional traits within the PTNs, Chl and LT were not connected to any other traits in the tree PTNs for either water–salt environment type. By contrast, the foliar C content was connected to other traits in all PTNs except for herbs in the low soil water–salt environment. This suggests that the foliar C content played a critical role in maintaining PTN stability in response to variation in soil moisture and salinity for most focal plant species.

The structure of the PTN varied among plant life forms and between the high and low soil water–salt environments (Table 4). In the high soil water–salt environment, the shrub PTN showed relatively high modularity (MD), as well as the highest density (D) and clustering coefficient (CC) of all PTNs. The highest MD and lowest D were seen for the tree PTN, while the herb PTN exhibited intermediate values for all three parameters. In a low soil water–salt environment, the shrub PTN again had the highest D and CC but a lower MD. The herb PTN had the highest MD but lower D and CC than PTN in the high soil water–salt environment. The tree PTN again had high MD, but the lowest CC of all life forms (Table 4).

### 2.3. Soil Factors Mediated the Structure of Plant Trait Networks

**Figure 2:**
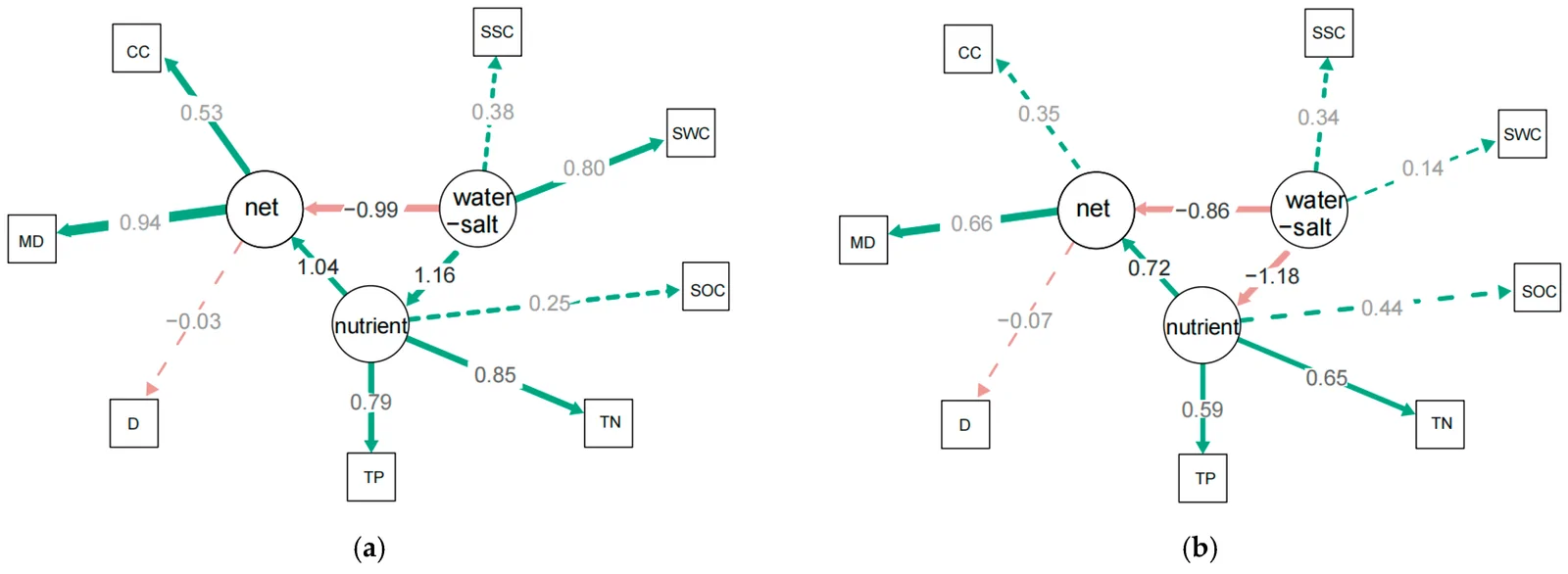
Structural equation models (SEMs) examining connections among soil factors and plant trait network structure for high (**a**) and low (**b**) soil water–salt environments. Circular nodes are latent variables, and square nodes are manifest variables. The “nutrient” latent variable includes the soil organic carbon (SOC), total nitrogen (TN), and total phosphorus (TP), while the “water–salt” latent variable includes the soil salt content (SSC) and soil water content (SWC); the “net” latent variable captures network parameters from the plant trait networks, including the clustering coefficient (CC), density (D), and modularity (MD). Solid green lines show positive effects, while solid red lines show negative effects; dashed lines indicate weak or nonsignificant effects. The values on the lines are path coefficients (quantifying the strength of each effect).

In a high soil water–salt environment, structural equation modeling (Figure 2) revealed that soil water–salt factors (represented by the latent variable “water–salt”) had a strong positive effect on overall soil nutrient availability (“nutrient”, path coefficient = 1.16), which was strongly associated with soil TN and TP. In turn, nutrient factors positively regulated overall PTN structure (as represented by the “net” latent variable, path coefficient = 1.04) and effectively improved network complexity. Meanwhile, soil water–salt factors had a direct negative effect on PTN structure (path coefficient = −0.99), indicating that water–salt imbalances would reduce PTN stability.

In a low soil water–salt environment, the connection between SOC and other nutrient factors was relatively weak in the structural equation model. The positive effect of nutrient factors on PTN structure was also lower (path coefficient = 0.72) than in the high water–salt environment model. Water–salt factors had a stronger negative effect on PTN structure (path coefficient = −0.86), indicating a greater constraint on PTN structure. In addition, water–salt factors negatively affected nutrient factors (path coefficient = −1.18), leading to a clear trade-off between moisture availability/salinity and nutrient supply, which further influenced PTN stability.

## 3. Discussion

### 3.1. Plant Trait Network Structure Differed Among Herbs, Shrubs, and Trees

Herbs, shrubs, and trees possess distinct ecological functions and strategies for resource acquisition. Hence, in combination with soil factors, plant life form was found to shape PTN topology [7]. Herbs adopted highly flexible survival strategies and could easily adjust key traits as needed, while shrubs maintained stable and highly coordinated trait associations over time; trees relied on networks with low connectivity and high modularity to buffer against abiotic stress. High PTN modularity allows different trait clusters (or modules) to respond to drought and salinity stress relatively independently. When faced with combined drought–salinity stress, individual modules can adjust independently without disrupting the overall network, thus avoiding sudden functional collapses; this helps ensure that desert ecosystems remain productive and stable over the long term [11]. Moreover, the unique PTNs of different life forms foster functional complementarity within desert plant communities, enhancing ecosystem resistance to environmental fluctuations [27]. Thus, two core adaptive mechanisms contribute to the functional differentiation of desert plant life forms: fine-tuning single traits within the leaf economic spectrum and large-scale restructuring of overall PTN topology [7].

In a high soil water–salt environment, the herb PTN exhibited high CC and D values, with LT serving as the central network trait. Thick leaves serve as a critical salt-tolerance trait; they balance photosynthesis with water retention needs to reduce osmotic stress under saline conditions [17,28,29]. Network MD was significantly higher for the low versus high soil water–salt environment, with the central trait shifting from LT to the N:P ratio. This shift likely represents a plastic survival mechanism. High LT prioritizes osmotic resistance in saline habitats, while adjustments to the N:P ratio can maximize growth under low-resource conditions. By flexibly replacing central network traits, desert herbs may rapidly switch resource allocation strategies to cope with fluctuating soil conditions, as shown by other studies [30,31].

In stark contrast to the flexible strategy used by herbs, shrubs had stable, tightly integrated trait networks that functioned effectively in both high and low water–salt environments. In a low soil water–salt environment, shrub PTNs had high CC and D values alongside low MD. Strong correlations among traits can elevate overall resource-use efficiency and buffer against abiotic stress, representing a stable, long-term stress-tolerance strategy. Such highly integrated PTNs have also been described for shrubs in arid ecosystems in northwest China [32], suggesting a widespread adaptive strategy utilized by dryland woody species. Shrub PTNs often include leaf N or the C:N ratio as central traits, sustaining steady trait coordination. In this study, shrub abundance was higher, and shrub PTNs were more densely connected, in a low soil water–salt environment, likely an adaptive response to heightened resource limitation. Scarce soil resources drive tighter correlations among functional traits and multi-dimensional resource trade-offs, thereby facilitating the more efficient capture and internal mobilization of limited water and nutrient resources [33].

In this study, trees often adopt a conservative strategy to mitigate the adverse effects of drought and salinity stress [34], showing PTNs with low connectivity (CC, D) and high MD. Modular trait networks have been widely reported for subtropical forests [35], as this architecture improves stress resistance and sustains long-term resource-use efficiency. As shown by other studies of arid zones, high PTN modularity acts as a key adaptive strategy in the face of resource scarcity and environmental heterogeneity. In the saline desert ecosystems examined here, modular network structure represented a unique long-term survival strategy exclusive to trees [17]. The greater modularity of tree PTNs suggests greater functional differentiation and stronger trait coordination. Five functional modules are often recognized: nutrient element accumulation, nutrient uptake, phenotypic structure, photosynthesis, and water regulation. These modules reveal the complexity of trait interactions and their central role in regulating plant growth and metabolism [36]. Overall, PTN differences among the three life forms illustrate how both environmental conditions and plant growth forms jointly shape network topology for desert plants [37]. Under abiotic stress, PTNs undergo plastic restructuring, with these responses varying among plant life forms [38].

### 3.2. Desert Plant Trait Networks Are Shaped by Soil Environmental Variation

Plant growth and development are closely associated with an individual’s local environment, and resource-use strategies are significantly moderated by habitat quality [10]. Variation in soil moisture and salinity was a key driver of PTN topology for desert plants in this study. In high water–salt environments, most network parameters (i.e., CC, D, and MD) for herb and tree PTNs were higher than in low water–salt environments, except for CC and D in shrub PTNs, where the reverse was true. Soil TN and TP were key drivers of network structure. These represent the most common limiting nutrients for plant growth in terrestrial ecosystems [39,40], and higher soil TN and TP can therefore enhance network connectivity and local clustering [38]. This partly explains the more complex network structure observed here in a high soil water–salt environment. These results suggest that resource-abundant habitats, with sufficient soil moisture and nutrients, allow plants to develop more complex and tightly coupled trait networks. Strengthening functional trait interactions further improves resource-use efficiency, enabling plants to adapt to combined drought and salinity stress [11].

Compared with a high soil water–salt environment, the low soil water–salt environment was relatively impoverished, having less soil moisture and nutrients. In the low soil water–salt environment, SWC and SSC negatively impacted soil TN and TP, indicating that nutrient accumulation was inhibited in the soil; this ultimately weakened the positive effects of soil nutrients on PTN parameters. Drought limits nutrient supply by restricting mineralization and reducing nutrient diffusion and mass flow in the soil; thus, decreases in soil moisture further impair plant nutrient uptake [8]. Under drought, plants may enhance their resource retention capacity by simplifying trait associations, ultimately leading to PTN structures with low connectivity and synergy [41,42]. Meanwhile, water and salt stress both tend to increase leaf mesophyll cell density, resulting in thicker and smaller leaves [43]; this is consistent with the greater LT and smaller LA observed for low versus high soil water–salt environments in this study. The decrease in PTN connectivity (indicating greater constraints on trait combinations) from high to low soil water–salt environments likely reflects an adaptive survival strategy for plants responding to decreases in moisture and nutrient availability [24,38].

By constructing PTNs for communities situated along soil moisture and salinity gradients, this study revealed resource utilization strategies used by different plant life forms and how drought and salinity stress shape adaptive traits. Central network traits may be used to identify salt- and drought-resistant native species, while network differences among life forms will inform restoration efforts for arid ecosystems. However, three study limitations should be considered. First, hydraulic, physiological, and root traits were not measured due to practical limitations associated with the field survey. By integrating these data in the future, aboveground–belowground trait networks could be constructed to better interpret whole-plant resource allocation patterns under abiotic stress. Second, adaptive strategies were inferred from PTN topology and lacked direct mechanistic verification. Multi-dimensional physiological measurements will be required to provide empirical evidence for these strategies. Third, the tree life-form group only contained *Populus euphratica* with limited sample size; we cannot disentangle species-specific effects from life-form effects, and tree-related findings are restricted to *P. euphratica* and should not be generalized to all desert tree species. In addition, as sampling was confined to a single desert region, the study findings cannot necessarily be extrapolated to other desert ecosystems. Cross-regional field surveys are needed to assess the universality of the trait coordination strategies uncovered here, as well as to better understand the underlying mechanisms.

## 4. Materials and Methods

### 4.1. Overview of the Study Area

The Ebinur Lake Wetland National Nature Reserve is situated in northwestern Jinghe County, Bortala Mongolian Autonomous Prefecture, Xinjiang, China (44°30′–45°09′ N, 82°36′–83°50′ E). It serves as a hub for the convergence of water and salt and is located at the lowest point along the southwestern margin of the Junggar Basin. The Reserve experiences a typical temperate continental desert climate, with year-round aridity. The average annual temperature is 7.8 °C, while recorded maximum and minimum temperatures are 41.3 °C and −36.4 °C, respectively. The annual precipitation averages 105.17 mm, compared with an annual evaporation of 2221.3 mm [31]. The Aqikesu River, which flows through the study area and is located on the eastern side of Ebinur Lake, is one of the lake’s water sources. The unique ecosystem surrounding Ebinur Lake supports diverse desert plants, including various herb, shrub, and tree species. Common herbaceous species include *Phragmites australis*, *Suaeda prostrata*, *Glycyrrhiza uralensis*, *Salsola collina*, *Suaeda microphylla*, *Salsola aperta*, and *Agriophyllum squarrosum*, while shrubs include *Karelinia capsica*, *Apocynum venetum*, *Alhagi sparsifolia*, *Suaeda salsa*, *Reaumuria songarica*, *Nitraria tangutorum*, *Kalidium foliatum*, *Halimodendron halodendron*, *Halostachys capsica*, *Halocnemum strobilaceum*, *Salsola ruthenica*, *Calligonum mongolicum*, and *Horaninowia ulicina*. Tree species include *Populus euphratica*, *Haloxylon ammodendron*, and *Tamarix ramosissima*. In recent years, climate change and human activities have become threats to local riparian vegetation [8].

### 4.2. Research Methods

#### 4.2.1. Field Survey and Plant Trait Measurements

Within the Ebinur Lake Wetland National Nature Reserve, the study area was situated on the north bank of the Aqikesu River. The vegetation in this location is typical of desert regions, with a stable climate throughout. The topography is relatively flat, with minimal elevation variation (between 290 and 310 m). In addition, the study area experiences little human disturbance or regular human activity. Previous field surveys have documented prominent soil moisture and salinity gradients that run perpendicular to the Aqikesu River within the study area [8]; in this study, plant communities and soil conditions were surveyed along these natural gradients.

Three transects (each ~1 km in length) were established perpendicular to the Aqikesu River, with an interval of 1 km between adjacent transects. Sampling points were set every 0.1 km along each transect, and three 10 m × 10 m quadrats were established at each sampling point, with a spacing of ~100 m between quadrats. Therefore, a total of 30 sampling points and 90 quadrats (30 × 3) were established across the three transects. To characterize soil moisture, nutrients, and salinity in the quadrats, soil samples (0–15 cm depth) were collected from three randomly selected bare-soil spots per quadrat, thoroughly mixed, and stored in self-sealing bags for later analysis.

Eight focal species (Table 2) were selected based on their abundance in the focal desert ecosystem. Study species were widely distributed within the study area, accounted for a large proportion of total abundance, and constituted essential components of the plant community. Three individuals were randomly selected per target species for morphological measurement, and their height, crown width, and basal diameter were measured. From each individual, three healthy, intact mature leaves of comparable size were obtained for functional trait measurements.

For the collected leaves, the relative chlorophyll content (Chl) was determined using a SPAD-502 chlorophyll meter (Konica Minolta, Osaka, Japan), with repeated measurements conducted on each leaf and then averaged to minimize measurement error. Leaf thickness (LT) was measured near the center of the leaf (avoiding the main vein) with a vernier caliper (precision: 0.01 mm), and the average value of several measurements was again taken as individual leaf thickness. Next, leaves were labeled, flattened with a transparent plastic sheet, and photographed with a ruler to determine the leaf area (LA). All raw trait data were field-checked by two researchers to eliminate data entry errors. Outliers falling more than 3 standard deviations from the mean of a given trait were excluded from the analysis to reduce interference from extreme individual samples. Similarly, samples with missing data or damaged leaves were removed from the dataset (without data interpolation).

Roughly 20 g of leaf tissue was collected for each sampled individual (three per species), placed in labeled envelopes, and returned to the laboratory for subsequent analysis. The leaf samples were dried in an oven at 75 °C for 48 h, ground into powder, and sieved prior to determining the carbon (C), nitrogen (N), and phosphorus (P) content. The foliar organic carbon content was measured using the potassium dichromate dilution and heating method, while the total nitrogen content was quantified via the Kjeldahl method (after H_2_SO_4_-H_2_O_2_ digestion); the total phosphorus content was determined using the molybdenum–antimony anti-colorimetric method (after H_2_SO_4_-H_2_O_2_ digestion) [44].

#### 4.2.2. Soil Sample Analysis

The soil water content (SWC) was measured by oven drying, while the soil salt content (SSC) was determined using electrical conductivity. As with the leaf samples, soil organic carbon (SOC) was assessed using the potassium dichromate dilution and heating method, soil total nitrogen (TN) via the Kjeldahl method, and soil total phosphorus (TP) using the HClO_4_-H_2_SO_4_ molybdenum–antimony anti-colorimetric method [44].

#### 4.2.3. Data Analysis

##### Plant Trait Networks

A matrix A = [a_i,j_] of Pearson’s correlation coefficients was first constructed among all measured plant traits. Plant traits were treated as nodes, and the correlations between them as edges. Herein, a_i,j_ ϵ [0, 1] was generated by assigning a value of one to correlations exceeding the significance threshold and zero to those below it. We performed sensitivity analyses using three correlation thresholds (|r| ≥ 0.1, 0.2, and 0.3) with *p* < 0.05, and the|r| > 0.2 with *p* < 0.05 threshold was finally selected for network construction [11,17,45]. The topological structure of these relationships was visualized using plant trait networks (PTNs) constructed in R. To describe network structure, PTN parameters were calculated using the igraph package (v1.3.4) in R (v4.3.1). Three key topological parameters were used to quantify PTN topology. (I) Density (D) represents the degree of aggregation among all nodes in the network; higher edge density indicates stronger synergistic relationships between traits, with increased connectivity facilitating more efficient resource acquisition and allocation in plants. (II) Modularity (MD) describes the degree of differentiation among sub-networks (or modules) within the PTN. Greater network modularity reflects stronger internal connectivity within modules, fewer connections between modules, and lower network complexity. Greater modularity may benefit plants in highly variable environments, where abiotic stressors tend to affect only specific traits and not others. Note that network metrics were not validated via resampling in the present analytical workflow. (III) The clustering coefficient (CC) describes how nodes are connected to neighboring nodes. It represents an average value for all traits, measuring the average probability that a given trait is connected to adjacent traits. If a node is fully connected to its neighbors, the clustering coefficient is one. A value close to zero signifies few connections with neighbors. Traits with high CC values represent key nodes within specific functional modules of the PTN [11].

##### Statistical Analyses

Before exploring the effects of soil factors on PTNs, k-means cluster analysis was used to categorize all study plots into two distinct groups. Plots with relatively high soil water and salt contents experienced heightened salt stress and were classified as “high soil water–salt environment”, whereas plots with relatively lower values were designated as “low soil water–salt environment”. Prior to fitting structural equation models (SEMs), multicollinearity was assessed among the soil variables. The variance inflation factor (VIF) was calculated using the vif() function in R. Variables with VIF > 10 would be removed. All environmental variables had VIF < 10 in both groups, so all five were retained for subsequent analyses [46]. Structural equation modeling was performed to disentangle the direct and indirect effects of soil factors on PTN topological parameters, with separate models developed for the high and low soil water–salt environments. Within the SEM framework, the soil water and salt content were set as exogenous variables, soil nutrient factors as mediating variables, and PTN topological parameters as endogenous variables. Path coefficients were estimated to quantify the magnitude and direction of causal effects, and all SEMs were constructed using the lavaan package (v0.6-17) in R.

## 5. Conclusions

This study found that PTN structure differed among herb, shrub, and tree species in a typical desert ecosystem, elucidating their divergent adaptation strategies to abiotic stress. For example, the central trait in the PTN differed among plant life forms. Herbs (central traits: leaf thickness and the N:P ratio) had high-density PTNs that revealed strong adaptability, allowing herbaceous plants to quickly adjust to environmental change. For shrubs (central traits: C and P), PTN connectivity was stable across soil environments, supporting overall plant community stability. Finally, trees (central traits: N and the C:P ratio) had highly modular PTNs, which suggests strong resistance to disturbance. Therefore, different life forms had distinct ecological functions: herbaceous plants promoted rapid resource turnover, shrubs promoted community stability, and trees promoted long-term stress resistance. Importantly, soil moisture, nutrient availability, and salinity also jointly regulated PTN topology. In a high soil water–salt environment, soil TN and TP were the key factors affecting PTN topology; in a low soil water–salt environment, SSC and SWC negatively affected PTN structure, increasing the difficulty of plant adaptation. In conclusion, PTN topology reflected desert plant adaptations to drought and salinity stress, providing crucial insights for the conservation of desert plant communities.

## Figures and Tables

**Table 1:** Comparison of soil properties between high and low soil water–salt environments. Different letters indicate significant differences between soil environments (*p* < 0.05).

Soil Water–Salt Environment	Soil Water Content	Soil Salt Content	Soil Organic Carbon	Total Nitrogen	Total Phosphorus
	(%)	(g/kg)	(g/kg)	(g/kg)	(g/kg)
High	7.92 ± 3.12 a	10.14 ± 4.66 a	4.41 ± 2.51 a	1.19 ± 0.66 a	0.58 ± 0.10 a
Low	3.83 ± 1.26 b	4.74 ± 1.25 b	3.09 ± 1.79 b	0.67 ± 0.34 b	0.49 ± 0.06 a

**Table 2:** Description of the eight focal plant species surveyed in this study.

Life Form	Plant Species Name	Family	Genus	Abundance	
				High Water–Salt	Low Water–Salt
Herb	*Glycyrrhiza uralensis*	Fabaceae	*Glycyrrhiza*	296	10
	*Phragmites australis*	Poaceae	*Phragmites*	2551	2247
Shrub	*Nitraria tangutorum*	Zygophyllaceae	*Nitraria*	60	205
	*Apocynum venetum*	Apocynaceae	*Apocynum*	1176	1211
	*Alhagi sparsifolia*	Fabaceae	*Alhagi*	254	636
	*Halimodendron halodendron*	Fabaceae	*Halimodendron*	100	0
	*Karelinia capsica*	Asteraceae	*Karelinia*	257	246
Tree	*Populus euphratica*	Salicaceae	*Populus*	53	26

**Table 3:** Comparison of plant functional traits between high and low soil water–salt environments, with trait values representing averages across all sampled individuals (belonging to eight herb, shrub, and tree species). Different letters indicate significant differences between soil environments (*p* < 0.05).

Plant Functional Trait	Units	Soil Water–Salt Environment	
		High	Low
C	g/kg	465.88 ± 30.22 a	460.12 ± 28.43 a
N	g/kg	20.10 ± 7.25 a	17.04 ± 5.94 b
P	g/kg	1.26 ± 0.24 b	1.37 ± 0.38 a
C:N	-	26.95 ± 9.54 b	31.04 ± 10.62 a
C:P	-	389.06 ± 70.58 a	368.75 ± 78.48 b
N:P	-	16.79 ± 6.67 a	14.05 ± 6.57 b
LT	mm	0.39 ± 0.14 b	0.48 ± 0.18 a
Chl	mg/g	49.01 ± 7.74 a	48.95 ± 8.69 a
LA	cm^2^	7.60 ± 8.27 a	7.25 ± 9.00 a

**Table 4:** Comparison of plant trait network topology among plant life forms and between high and low soil water–salt environments.

Soil Water–Salt Environment	Plant Life Form	Modularity	Density	Clustering Coefficient
High	Herb	0.106	0.472	0.639
	Shrub	0.114	0.556	0.714
	Tree	0.167	0.25	0.632
Low	Herb	0.278	0.333	0.621
	Shrub	0.019	0.694	0.884
	Tree	0.161	0.306	0.161

## Data Availability

The original contributions presented in the study are included in the article, further inquiries can be directed to the corresponding author.

## Abbreviations

The following abbreviations are used in this manuscript:

PTN	Plant trait network
MD	Modularity
D	Density
CC	Clustering coefficient
